# Nutrition, Inflammation, and Infectious Disease

**DOI:** 10.1155/2012/172480

**Published:** 2012-05-13

**Authors:** Helieh S. Oz, Sung-Ling Yeh

**Affiliations:** ^1^Departments of Physiology and Internal Medicine, College of Medicine, University of Kentucky Medical Center, KY, USA; ^2^School of Nutrition and Health Sciences, Taipei Medical University, Taipei 110, Taiwan

Pathogens and their related toxins can stimulate the production of endogenous mediators leading to dysregulated reactive oxidative stress, inflammatory responses, and alterations of organ functions. Nutrition support is necessary to protect against infectious and inflammatory conditions, but the optimal nutritional formulation is still under investigation.

This special issue focuses on 5 distinct papers to deal with the pathogens and their products (i.e.,* H. pylori, LPS*), specific nutrients (i.e., arginine, iron, plant extracts, antioxidants), and intervention and mechanism of interaction between various biological modifiers (i.e., superfamily of cytokines, inflammatory markers, adhesion molecules) involved in regulating the immune responses.

**H. pylori and Iron Loss**

*H. pylori *is an important pathogen in the etiology of chronic gastritis and intestinal metaplasia. Studies suggest a link between iron deficiency anemia and *H. pylori* infection. However, the mechanism of action by which *H. pylori* causes anemia is yet to be defined. One possible mechanism is that lactoferrin captures the iron from transferrin mediated by the bacterial outer layer receptor to initiate iron loss in the feces. In a pilot clinical trial, Y. Doğan and his collogues seek to determine the lactoferrin levels as a marker of anemia in *H. pylori *patients by means of immunohistochemical staining.

**Magnolia to Preserve Bones**

Skeletal structure turnover is organized by multifactorial regulators including cytokines and chemokines. In this process, osteoblasts release the osteoclastogenetic molecules such as RANKL and TNF-*α* and IL-6. Here, Magnolol isolated from* Magnolia officinalis *is reported to significantly block the osteoclastic differentiation in osteoblast MC3T3-E1 cultures. Therefore, E. J. Kwak et al. propose that Magnolol to preserve osteoblasts and possibly to prevent bone resorption and osteoporosis.

**Zerumbone and Pancreatitis**

D. Wenhong et al. investigate the effects of zerumbone pretreatment on acute necrotizing pancreatitis. They suggest zerumbone to attenuate the severity of pancreatitis as well as pancreatitis-induced hepatic injury through NF-*κ*B activation inhibition and downregulation of the ICAM-1 and IL-1*β*.

**Arginine Deiminase and IBD**

Arginine, a conditional amino acid, is synthesized in the normal cells and tissues. Arginine deiminase (ADI) is a key enzyme player in the cell signaling pathways, apoptosis, differentiation, and transcriptional regulation. ADI function is dysregulated in multiple human inflammatory diseases and cancer. Since certain tumor cells and pathogens require arginine for growth, selective elimination of arginine may act as a cancer therapy and is being investigated in human trials. While ADI is very immunogenic with a short lifespan, when covalently modified by polyethylene glycol (ADI-PEG), it becomes more stable and less antigenic. H. S. Oz et al. challenge 2 diverse murine models representing Crohn's disease and ulcerative colitis to demonstrate the protective action of this novel microbial enzyme in attenuating inflammatory responses by suppressing macrophage infiltration and iNOS expression.

**Prediction of Survival in Sepsis**

Finally, septic shock and multiple-organ dysfunction syndrome (MODS) are associated with 40–70% mortality rate in the intensive care units. T. Brenner et al. study the dysfunction of the arginine/nitric-oxide pathway in the pathogenesis of MODS and septic shock patients using an ELISA diagnostic technique. This simple technique may serve as a tool to predict the survival specifically in the severe sepsis patients with acute hepatic failure.

*Helieh S. Oz* *Helieh S. Oz* *Sung-Ling Yeh* *Sung-Ling Yeh*

